# Problems with Applying the Ozawa–Avrami Crystallization Model to Non-Isothermal Crosslinking Polymerization

**DOI:** 10.3390/polym14040693

**Published:** 2022-02-11

**Authors:** Sergey Vyazovkin, Andrey Galukhin

**Affiliations:** 1 Department of Chemistry, University of Alabama at Birmingham, 901 S. 14th Street, Birmingham, AL 35294, USA; 2 Alexander Butlerov Institute of Chemistry, Kazan Federal University, 18 Kremlevskaya Street, 420008 Kazan, Russia

**Keywords:** activation energy, calorimetry, curing, kinetics

## Abstract

Ozawa has modified the Avrami model to treat non-isothermal crystallization kinetics. The resulting Ozawa–Avrami model yields the Avrami index (*n*) and heating/cooling function (*χ*(*T*)). There has been a number of recent applications of the Ozawa–Avrami model to non-isothermal crosslinking polymerization (curing) kinetics that have determined *n* and have used *χ*(*T*) in place of the rate constant (*k*(*T*)) in the Arrhenius equation to evaluate the activation energy (*E*) and the preexponential factor (*A*). We analyze this approach mathematically as well as by using simulated and experimental data, highlighting the following problems. First, the approach is limited to the processes that obey the Avrami model. In cases of autocatalytic or decelerating kinetics, commonly encountered in crosslinking polymerizations, *n* reveals a systematic dependence on temperature. Second, *χ*(*T*) has a more complex temperature dependence than *k*(*T*) and thus cannot produce exact values of *E* and *A* via the Arrhenius equation. The respective deviations can reach tens or even hundreds of percent but are diminished dramatically using the heating/cooling function in the form [*χ*(*T*)]^1/*n*^. Third, without this transformation, the Arrhenius plots may demonstrate breakpoints that leads to questionable interpretations. Overall, the application of the Ozawa–Avrami model to crosslinking polymerizations appears too problematic to be justified, especially considering the existence of well-known alternative kinetic techniques that are flexible, accurate, and computationally simple.

## 1. Introduction

The Avrami model (also known as the Johnson–Mehl–Avrami–Kolmogorov model [1,2]) has been developed to describe the kinetics of the crystalline phase formation via the nucleation mechanism. The most common application area of the model is the kinetics of crystallization [1,2]. The classical Avrami model applies to isothermal conditions and is typically used in the following form:

(1) $$1-\alpha =\exp\left[-k_{A}\left(T\right)t^{n}\right]$$

where *t* is the time, *α* is the fractional volume transformed into the crystalline phase, *n* is the Avrami exponent (or index), and *k_A_*(*T*) is the Avrami rate constant.

An essential feature of the model is that it imitates the so-called sigmoid kinetics, i.e., the situation when the process rate initially increases, reaches a maximum, and finally decreases. Respectively, the cumulative growth of the new phase demonstrates a sigmoid profile with respect to time. It is noteworthy that apart from crystallization, there are a multitude of processes that demonstrate the sigmoid kinetics. This fact has inspired a variety of applications of the Avrami model that have stretched far beyond the process of crystallization. Some examples include: gelation [3,4,5], the adsorption of solutes [6,7,8] and gases [9,10], solution phase separation [11], aggregation and precipitation [12], and even the spread of new consumer products in the social networks [13].

There has also been a consistent interest in applying the Avrami model to the kinetics of crosslinking polymerization. The earliest works date back to the 1970s and 1980s [14,15]. Currently, this approach has been utilized in a number papers that have appeared to be inspired by a series of publications by Lu et al. [16,17,18,19]. All these publications have dealt with the isothermal kinetics and are thus beyond the scope of the present paper. Here, we focus exclusively on the non-isothermal kinetics of crosslinking polymerization as treated by the Avrami model modified by Ozawa [20]. We call this modification the Ozawa–Avrami model. In his original publication, Ozawa describes a method of adjusting the Avrami model to non-isothermal conditions so that it can be applied to describe the crystallization of polymers. The method yields the so-called heating or cooling function and the Avrami exponent [20]. Ozawa makes no mention of using this function for determining the activation energy. The idea of such a usage takes its origin from a paper by Lu et al. [21] and is actively being pursued by other scholars [22,23,24,25,26,27].

The objective of this study is to reveal the problems that can arise when applying the Ozawa–Avrami model to non-isothermal crosslinking polymerization. Generally, these problems are of two kinds. First, the Ozawa–Avrami model is inapplicable when the process kinetics is not of the sigmoid type. This is a significant limitation because the crosslinking polymerizations are known [28,29,30] to demonstrate autocatalytic (sigmoid) and reaction-order (decelerating) kinetics. Second, even if the process demonstrates autocatalytic kinetics, it is impossible to determine the correct values of the activation energy and preexponential factor from the heating/cooling function. These problems are illustrated by using both simulated (Section 4 and Section 5) and experimental (Section 6) data. The simulations are carried out by using the standard single-step models [31]. Although such an approach is employed most commonly, one should be aware of an advanced alternative that allows one to simulate crosslinking polymerization with an account of multiple reaction steps [32].

## 2. Avrami and Ozawa–Avrami Models

One of the most common techniques used to monitor crystallization is differential scanning calorimetry (DSC) and/or differential thermal analysis (DTA). The application is based on the fact that the volume of the crystalline phase that is formed is proportional to the heat released during crystallization.

In kinetic terms, it is assumed that the rate of crystallization is directly proportional to the heat flow *dQ*/*dt*:

(2) $$\frac{d\alpha }{dt}=\frac{1}{Q_{0}}\frac{dQ}{dt}=k\left(T\right)f\left(\alpha \right)=A\exp\left(\frac{-E}{RT}\right)f\left(\alpha \right)$$

where *Q*_0_ is the total heat released, *k(T)* is the Arrhenius rate constant, *E* is the activation energy, *A* is the preexponential factor, *R* is the gas constant, and *f*(*α*) is the reaction model. Note that because the heat flow is not species specific, the respective rate generally has an overall nature, i.e., it can correspond to more than a single reaction step. For this reason, the parameters of Equation (2) also generally have an overall nature.

The aforementioned proportionality between the rate and heat flow was introduced in an early work by Borchardt and Daniels [33]. Their analysis of the heat flow components as measured by DTA or heat flux DSC suggests that one can neglect the so-called thermal inertia term. This, however, may not always be justified. In particular, when one uses larger sample masses and faster heating rates, neglecting the thermal inertia term can result in significant systematic errors in the activation energy [34,35]. In this regard, it is important to stress that accounting for thermal inertia can be accomplished via a relatively simple procedure [35].

Per Equation (1), the extent of conversion is determined experimentally as the fractional area of the DSC peak. Then, the parameters of the Avrami model can be estimated from Equation (3)

(3) $$\ln\left[-\ln\left(1-\alpha \right)\right]=\ln k_{A}\left(T\right)+n\ln t$$

by plotting its left-hand side against the ln*t*. Assuming that *k_A_*(*T*) has an Arrhenian temperature dependence permits evaluating the Arrhenius parameters, i.e., *E* and ln*A* [36]. However, the direct substitution of *k_A_*(*T*) into the Arrhenius equation as follows:

(4) $$\ln k\left(T\right)=\ln A-\frac{E}{RT}$$

creates some problems with correctly estimating its parameters. The issue has been addressed in several publications [37,38,39,40,41]. Briefly, one needs to recognize that the rate in Equation (2) invariably has the units of (time)^−1^. Since *f*(*α*) is dimensionless, *k*(*T*) also has to have the units of (time)^−1^. However, estimating *k_A_*(*T*) from the Avrami equation (Equations (1) or (3)) yields the value that has the units of (time)^-n^. For that reason, it should not be substituted directly into the Arrhenius Equation (4). The problem is resolved by a simple transformation that converts the units of *k_A_*(*T*) into (time)^−1^:

(5) $$k\left(T\right)={\left[k_{A}\left(T\right)\right]}^{\frac{1}{n}}$$

Without this transformation, the direct substitution of *k_A_*(*T*) in Equation (4) results in estimating apparent Arrhenius parameters that are *n* times larger than the correct values. That is: [37,39,41]

(6) $$E_{A}=nE,\;\ln A_{A}=n\ln A$$

The problem is avoided altogether if the Avrami equation is used in the modified form: [37,38,39,40,41,42]

(7) $$1-\alpha =\exp\left[-{\left(k_{mA}\left(T\right)t\right)}^{n}\right]$$

The resulting Avrami rate constant, *k_mA_* always has the units of (time)^−1^, and thus, its direct substitution into Equation (4) yields the correct values of *E* and ln*A*. Overall, depending on the form of the Avrami equation used, the correct values of *E* and ln*A* are determined by defining the Arrhenius rate constant as follows:

(8) $$\ln k\left(T\right)=\frac{1}{n}\ln k_{A}\left(T\right)=\ln k_{mA}\left(T\right)$$

Ozawa has developed a method of adjusting the Avrami model for non-isothermal conditions [20]. The basic equation of the resulting Ozawa–Avrami model is:

(9) $$1-\alpha =\exp\left[\frac{-\chi \left(T\right)}{\beta^{n}}\right]$$

where *β* is the cooling or heating rate and *χ*(*T*) is, respectively, the cooling or heating function. The parameter *n* is sometimes confusingly called the Ozawa exponent, but in fact is the Avrami exponent [43]. The method makes use of the isothermal sections of the non-isothermal data. That is, one selects temperature and then determines what values of *α* correspond to this temperature at different values of *β*. Then, *n* and *χ*(*T*) are readily evaluated using Equation (10):

(10) $$\ln\left[-\ln\left(1-\alpha \right)\right]=\ln\chi \left(T\right)-n\ln\beta \;$$

by plotting the left-hand side against ln*β*.

Although Ozawa has never mentioned that *χ(T)* can be applied to estimating the Arrhenius parameters, such an application has been used in numerous publications devoted to the kinetic analysis of crosslinking polymerization [21,22,23,24,25,26,27]. In essence, they have proposed substituting *χ*(*T*) for *k*(*T*) in the Arrhenius Equation (4) to determine *E* and ln*A*. In our opinion, this is a questionable proposition. The first clue of this being in question is that *χ*(*T*) has units of (heating or cooling rate)^−n^, whereas *k*(*T*) of (time)^−1^, i.e., these are two different physical quantities. The actual relationship between *χ*(*T*) and *k*(*T*) is established to be: [44,45,46,47]

(11) $$\frac{d{\left[\chi \left(T\right)\right]}^{1/n}}{dT}=-{\left[k\left(T\right)\right]}^{1/n}$$

The derivations of Equation (11) are not presented here as they are readily available in the original publications [44,45,46,47]. Note that the negative sign in the right-hand side of Equation (11) applies only to the conditions of cooling, i.e., when *β* < 0. This is because on cooling *χ*(*T*) is a decreasing function of temperature [20], so its derivative is negative, whereas *k*(*T*) must always be positive. For the conditions of heating, the derivative is positive, so the negative sign is dropped. Clearly, *χ*(*T*) is nothing else but the rate constant integrated over temperature. Perhaps some empirical support for using *χ*(*T*) in the Arrhenius equation comes from the fact that ln*χ*(*T*) tends to show a reasonably linear dependence on *T*^−1^ and yields the *E* values rather similar to the activation energies. These issues are analyzed further by using some simulated data.

## 3. Simulations

To look closer into the use of *χ*(*T*) for estimating the Arrhenius parameters as well as the Ozawa–Avrami model in general, we simulate processes of two types: one that obeys the Avrami model with *n* = 3 and another that follows the second-order kinetics model. These models are commonly denoted as A3 and F2, respectively. Note that the reaction-order kinetics are routinely encountered in crosslinking polymerizations as seen in examples publicized elsewhere [28,29,30]. Both processes have the same Arrhenius parameters: *E* = 100 kJ mol^−1^ and *A* = 10^12^ min^−1^ (i.e., ln*A* = 27.63) and are simulated in the form of *α* vs. *T* curves at five heating rates: 1, 1.5, 2, 3, and 5 K min^−1^. This is accomplished by solving the following equation for *α*:

(12) $$g\left(\alpha \right)=\frac{A}{\beta }\int_{0}^{T}\exp\left(\frac{-E}{RT}\right)dT$$

where *g*(*α*) is ${\left[-\ln\left(1-\alpha \right)\right]}^{\frac{1}{3}}$ for the A3 model or ${\left(1-\alpha \right)}^{-1}-1$ for the F2 model [31,39,48]. The temperature integral is solved with the aid of a highly accurate approximation [49].

## 4. Analysis of Simulated A3 Data

**Figure 1:**
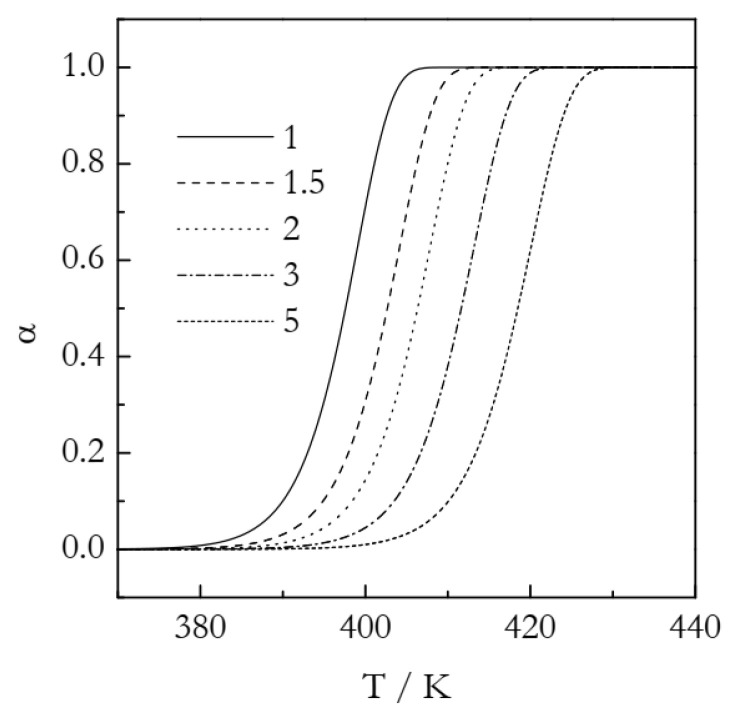
Simulated kinetic curves for the A3 process. Numbers by the lines are heating rates in K min^−1^.

**Figure 2:**
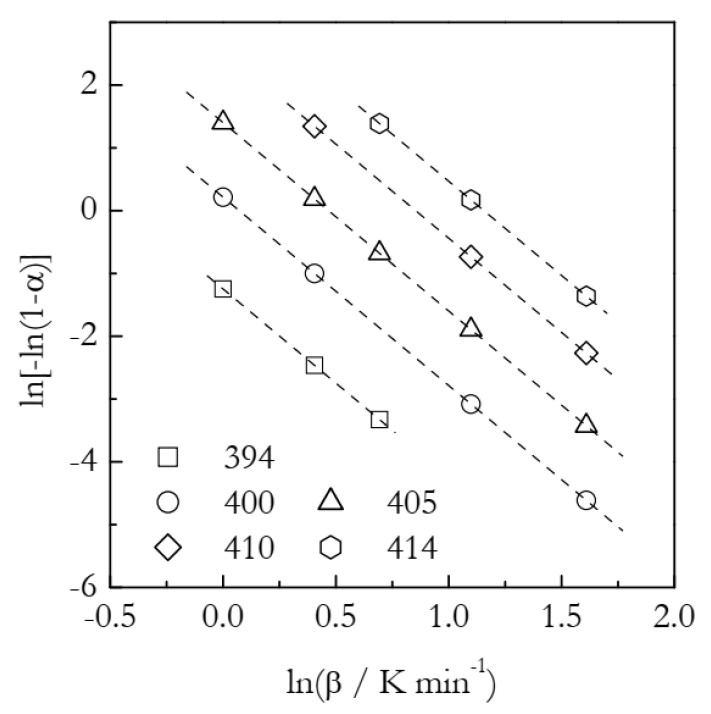
Ozawa plots for the A3 process. Numbers by the symbols are temperatures in K. Dash lines are least square fits to the respective points.

**Figure 3:**
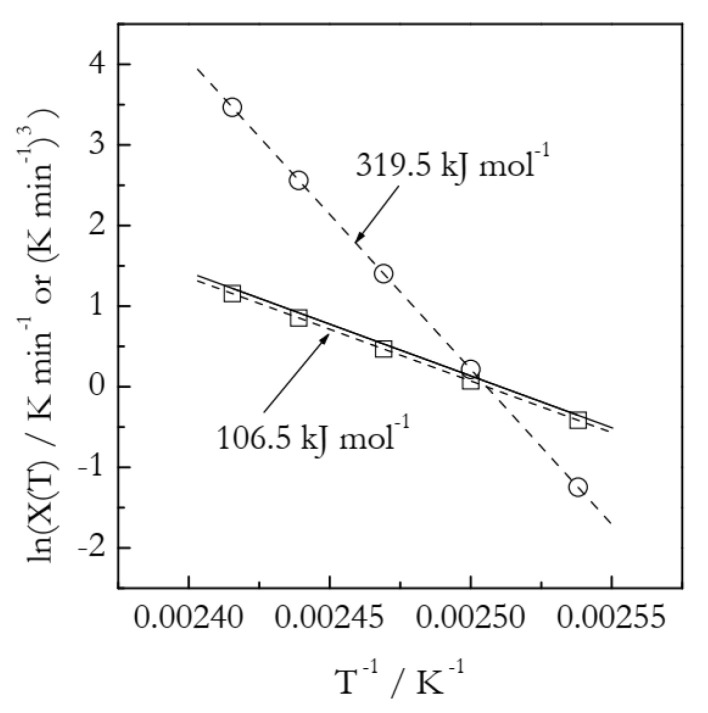
Arrhenius plots of the heating function. Circles and squares represent, respectively, ln*χ*(*T*) and ln*χ*(*T*)/*n* values; dash lines are corresponding least square fits. Solid line is plotted by substituting the correct values of *E* and *A* into Equation (15). Least square fit to the solid line yields *E* = 106.7 kJ mol^−1^.

The simulated kinetic curves are displayed in Figure 1. Figure 2 shows a set of the Ozawa plots (Equation (10)) obtained from the simulated A3 data. As expected, the plots are perfectly linear (the coefficient of linear correlation in all cases is 1) and yield the exact values of *n* (Table 1). These plots also permit estimating the ln*χ*(*T*) values. Indeed, the Arrhenius plots of ln*χ*(*T*) vs. *T*^−1^ demonstrate excellent linearity (Figure 3). The activation energy estimated directly from such a plot is 319.5 kJ mol^−1^, which is more than three times larger than the value used in simulations. The source of this large difference is probably the same as in the case of using ln*k_A_*(*T*) instead of ln*k_A_*(*T*)/*n* (see Equation (6)). Expectedly, plotting ln*χ*(*T*)/*n* vs. *T*^−1^ yields a three times smaller value, *E* = 106.5 kJ mol^−1^. It may seem to be reasonably close to the simulated value. The respective systematic error is 6.5%, which could be considered acceptable in the case of experimental data but not for the case of simulated data. For ln*A*, the error is even larger: 16%. At any rate, for the simulated data, we have to be obtaining the exact values. For comparison, the estimated *n* values are exact, at least to the third decimal place (Table 1). That is, the error is less than 0.1%. Thus, the errors in *E* and ln*A* should be expected to be on the level of a fraction of a percent.

Of course, the question that arises is “Why are we getting linear Arrhenius plots for the *χ*(*T*) although it represents a quantity that differs entirely from *k(T)*?” The basic reason is quite simple. The integration of the exponential function in *k(T)* (see Equation (11)) would necessarily include a similar exponential function in *χ(T)*, which simply is a property of the exponential function. To illustrate the point, consider the simplest case of *n* = 1. Then, for the conditions of heating, Equation (11) can be transformed into

(13) $$\chi \left(T\right)=A\int_{0}^{T}\exp\left(\frac{-E}{RT}\right)dT$$

The temperature integral in Equation (13) does not have an analytical solution. Yet, it has a number of accurate approximations [50], the simplest of which is as follows: [51]

(14) $$\chi \left(T\right)=\frac{ART^{2}}{E}\exp\left(\frac{-E}{RT}\right)$$

As we can see, the integrated form, *χ*(*T*), does include the same exponential function as the Arrhenius rate constant, *k*(*T*). Taking the logarithm of both sides of Equation (14), we obtain:

(15) $$\ln\left[\chi \left(T\right)\right]=\ln\frac{AR}{E}+2\ln T-\frac{E}{RT}$$

Let us now substitute the simulated (true) values of *E* = 100 kJ mol^−1^ and *A* = 10^12^ min^−1^ into Equation (15) and plot the ln*χ*(*T*) vs. *T*^−1^ dependence for the temperature range 394–414 K (i.e., the same temperature range that we used for the Ozawa analysis (Table 1)). In Figure 3, we can see that this dependence is practically the same as the dependence of ln*χ*(*T*)/*n* vs. *T*^−1^ plotted by using the data from Table 1. This obviously indicates that Equation (15) accurately imitates the behavior of the ln*χ*(*T*)/*n* vs. *T*^−1^ dependence obtained from the Ozawa analysis (Table 1). Indeed, fitting the ln*χ*(*T*) vs. *T*^−1^ dependence obtained by Equation (15) to a straight line yields practically the same values of the intercept and *E* as the corresponding values obtained by fitting the ln*χ(T)*/*n* vs. *T*^−1^ data from Table 1 to the Arrhenius equation (Equation (4); see Figure 3). The resulting absolute value of the coefficient of linear correlation for the Arrhenius plot set by Equation (15) is 1, just as for the two other plots in Figure 3.

In general, Equation (15) suggests that a strong linearity for ln*χ*(*T*)/*n* vs. *T*^−1^ data should be expected as long as the 2ln*T* term does not vary much with the temperature. In the temperature range 394–414 K, this term increases by only 0.8%, which is practically negligible. By taking the derivative of Equation (15) with respect to *T*^−1^, one can easily find that the *E* derived from *χ*(*T*) is larger than the true value by 2*RT*. That is, the relative systematic deviation of the activation energy estimated with the plot ln*χ*(*T*)/*n* vs. *T*^−1^ from the true value should increase for processes occurring at a higher temperature and with a lower activation energy. Furthermore, it should be stressed that one should not stretch the predictions of Equation (15) too far because the approximation used (Equation (14)) is reasonably accurate within the range 28 < *E*/*RT* < 50 [50].

To further illustrate the inaccuracy of using the *χ*(*T*) value for estimating the Arrhenius parameters, we treat the simulated A3 data with an advanced isoconversional method [52]. In it, the activation energy *E_α_* is found at multiple conversions with the step Δ*α* (usually 0.01 or 0.02). As a result, one obtains a dependence of *E_α_* on *α*. For each *α*, *E_α_* is found as a minimum of the function:

(16) $$\Psi \left(E_{\alpha }\right)=\overset{n}{\underset{i=1}{\sum }}\overset{n}{\underset{j\neq i}{\sum }}\frac{J\left[E_{\alpha },T_{i}\left(t_{\alpha }\right)\right]}{J\left[E_{\alpha },T_{j}\left(t_{\alpha }\right)\right]}$$

where the integral:

(17) $$J\left[E_{\alpha },T_{i}\left(t_{\alpha }\right)\right]\equiv \int_{t_{\alpha -∆\alpha }}^{t_{\alpha }}\exp\left[\frac{-E_{\alpha }}{RT_{i}\left(t\right)}\right]dt$$

is evaluated by using the trapezoid rule. The preexponential factor is then estimated by using the compensation effect equation [31,53]. The resulting dependencies of *E_α_* and ln*A_α_* on *α* have demonstrated the constancy of both values, as expected for a single-step process. The mean values are found to be *E* = 100.05 kJ mol^−1^ and ln*A* = 27.59. They deviate, respectively, from the exact values by 0.05 and 0.1%, as opposed to the 6.5 and 16% deviations found for *E* and ln*A* determined from using *χ*(*T*). The above examples clearly indicate that the expected accuracy of estimating *E* and ln*A* from using the model data should, respectively, be on the scale of a fraction of a percent, i.e., definitely not 6.5 and 16%.

## 5. Analysis of Simulated F2 Data

**Figure 4:**
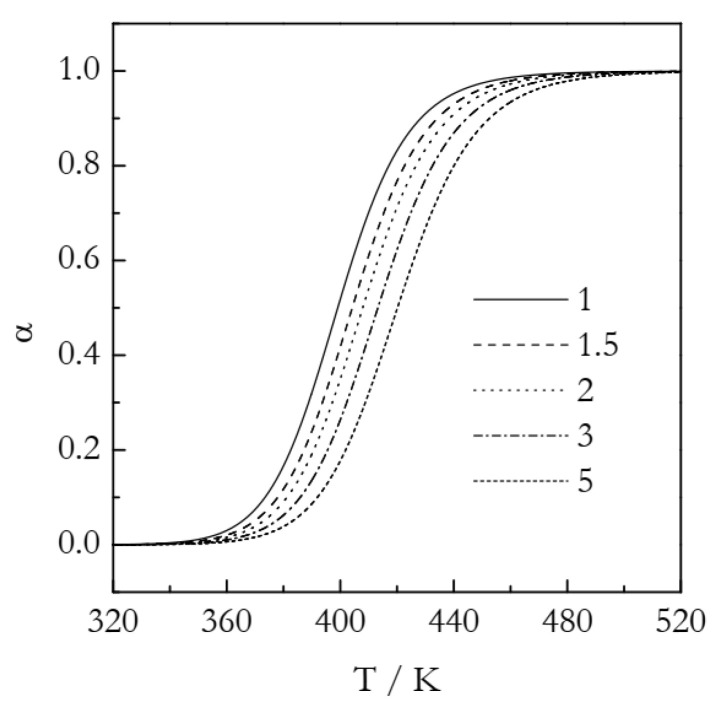
Simulated kinetic curves for the F2 process. Numbers by the lines are heating rates in K min^−1^.

**Figure 5:**
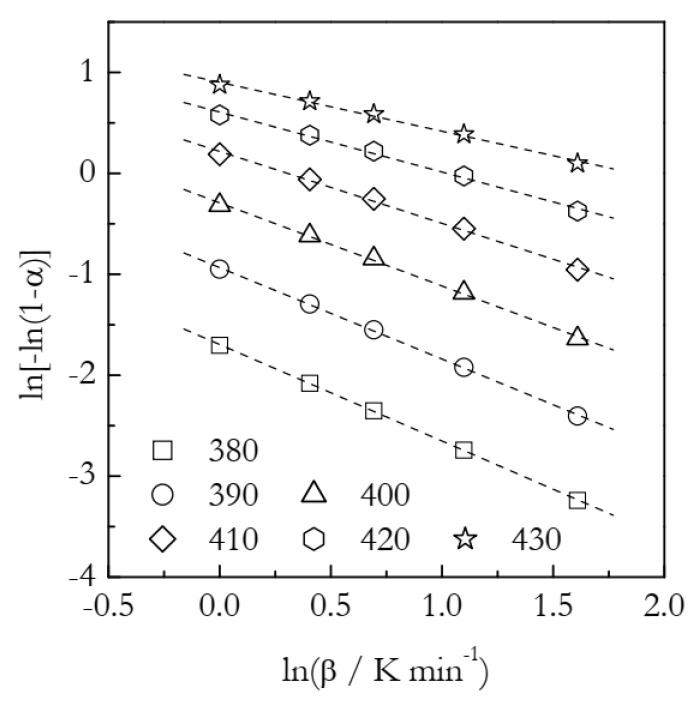
Ozawa plots for the F2 process. Numbers by the symbols are temperatures in K. Dash lines are least square fits to the respective points.

The simulated kinetic curves are displayed in Figure 4. Figure 5 presents a set of the Ozawa plots (Equation (10)) obtained from the simulated F2 data. The plots are nearly perfectly linear. The lowest coefficient of linear correlation is 0.997. An obvious difference between these plots and the ones for A3 is that the slope appears to change with temperature. Indeed, the values of *n* decrease systematically with temperature (Table 2). This is a clear indication that the Avrami model is inapplicable to the data that do not represent the sigmoid kinetics. It is noteworthy that a similar decrease in *n* with increasing temperature is found in experimental data on non-isothermal crosslinking [21,23,27], which thus may be a sign of the inapplicability of the Avrami model. Naturally, if the Avrami model does not apply to the data, one faces the problem of the applicability of the Ozawa–Avrami analysis altogether.

**Figure 6:**
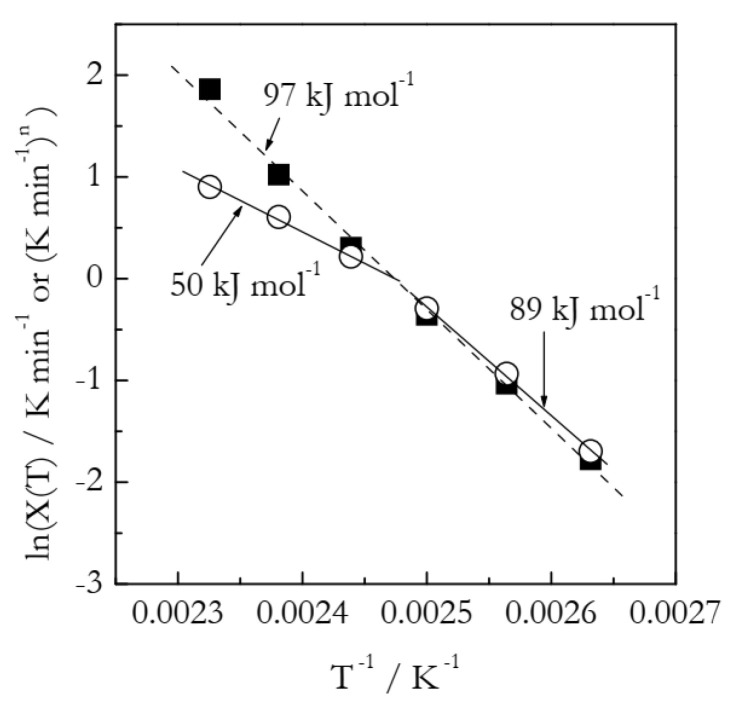
Arrhenius plots of the heating function. Circles and squares represent, respectively, ln*χ*(*T*) and ln*χ*(*T*)/*n* values. Two solid lines represent least square fits to three lower and higher temperature ln*χ*(*T*) values, respectively. Dash line is a least square fit to ln*χ*(*T*)/*n* values.

Figure 6 shows the Arrhenius plots built by employing the heating function data (Table 2). We should take notice that ln*χ*(*T*) vs. *T*^−1^ is distinctly nonlinear and thus can be described as a combination of two linear segments or, to put it differently, as an Arrhenius plot with a breakpoint. Such a situation is commonly interpreted as evidence of the reaction mechanism change. Of course, in the case of our simulated data, we know perfectly well that the process obeys a single mechanism with a single set of the Arrhenius parameters. Needless to say, the activation energy values, 89 and 50 kJ mol^−1^, estimated from the two linear segments (see Figure 6) deviate significantly from the simulated value 100 kJ mol^−1^. The importance of this example is that it clearly indicates that a breakpoint observed in the ln*χ*(*T*) vs. *T*^−1^ plot can be a trivial computational artifact. The latter can simply be due to the inapplicability of the Avrami model and can thus have no physical significance. Again, we should notice that the breakpoints have been customarily seen [21,23,25,26,27] in experimental ln*χ*(*T*) vs. *T*^−1^ plots for crosslinking.

As found in the case of the simulated A3 data, the Arrhenius parameters are better estimated by using the ln*χ*(*T*)/*n* vs. *T*^−1^ plots. The corresponding plot is depicted in Figure 6. It is remarkable that the aforementioned breakpoint does not appear in this plot. This is yet another clue that the breakpoint seen in the ln*χ*(*T*) vs. *T*^−1^ plot can be no more than a computational artifact. The plot is linear (the coefficient of linear correlation 0.9978), as expected. It yields the following Arrhenius parameters: *E* = 96.9 kJ mol^−1^ and ln*A* = 28.8. The respective deviations from the correct values are 3 and 10%. On the other hand, an advanced isoconversional method yields *E* = 100.2 kJ mol^−1^ and ln*A* = 27.88, which, respectively, deviate from the correct values by only 0.2 and 0.9%.

## 6. Analysis of Experimental Data

**Figure 7:**
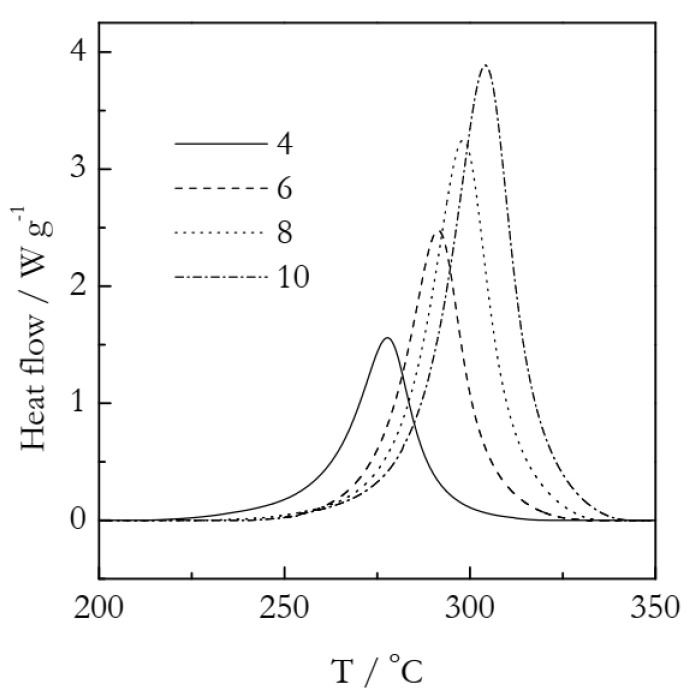
Experimental DSC curves for the polymerization of cyanate ester. Numbers by the lines are heating rates in K min^−1^. Adapted with permission from Galukhin et al. [54]. Copyright 2021 Wiley-VCH.

As an experimental example, we employ our previously published data on the crosslinking polymerization kinetics of cyanate ester based on a dimer of 4-tert-butylphenol [54]. Figure 7 shows the DSC curves representing this process. The details of the experiments and kinetic treatment are provided in a previous publication [54]. A comprehensive kinetic analysis has demonstrated that the process follows single-step kinetics. The Arrhenius parameters have been determined to be: *E* = 85 kJ mol^−1^ and ln(*A*/min^−1^) = 18.72. The kinetics of the process have been established to be autocatalytic, i.e., of the sigmoid type. The actual model is f(α) = α^1.20^(1 − α)^1.33^. As the kinetics are sigmoid, the Avrami model can be used as a reasonable approximation.

**Figure 8:**
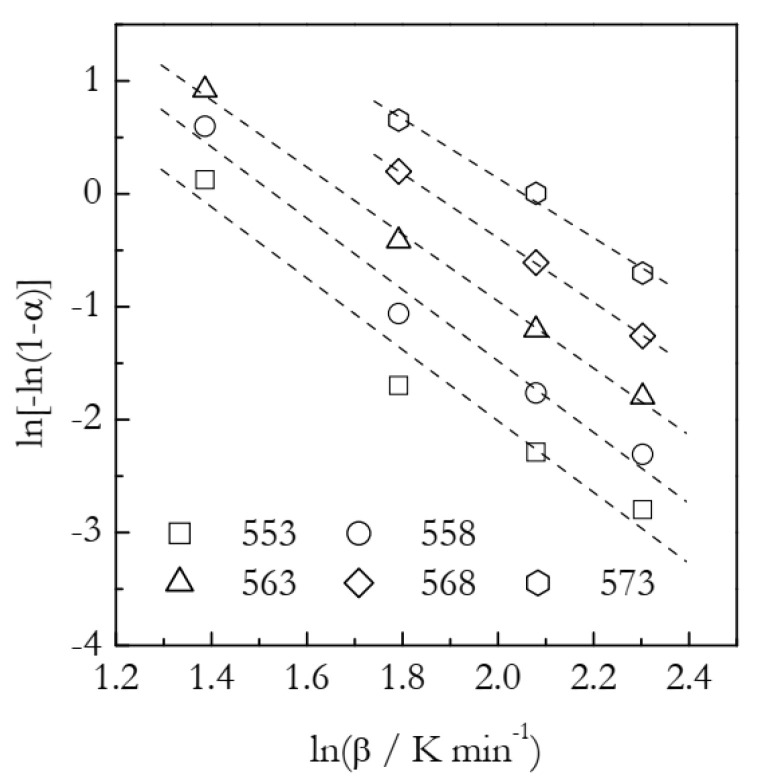
Ozawa plots for cyanate ester polymerization. Numbers by the symbols are temperatures in K. Dash lines are least square fits to respective points.

The Ozawa plots are displayed in Figure 8. The points appear to fall on the straight lines reasonably well. The *n* and *χ*(*T*) values derived from the fit lines are collected in Table 3. It is seen that the *n* values decrease systematically with an increasing temperature. Although the trend is not nearly as strong as in the case of the F2 data (Table 2), this still may be indicative that the Avrami model is not a proper representation of the process under study.

**Figure 9:**
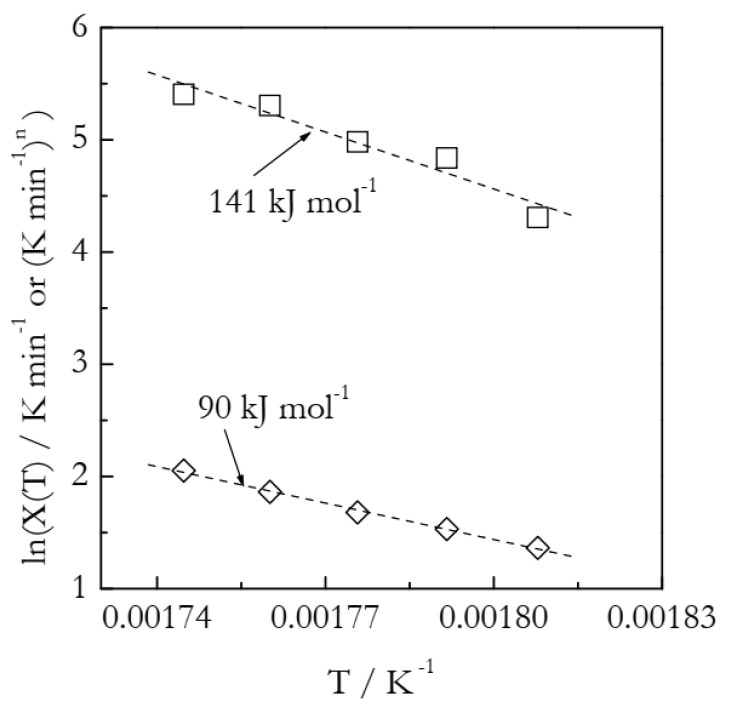
Arrhenius plots of the heating function. Squares and diamonds represent, respectively, ln*χ*(*T*) and ln*χ*(*T*)/*n* values. Dash lines are least square fits to the respective points.

Figure 9 presents the Arrhenius plots based on the heating function data (Table 3). The ln*χ*(*T*) vs. *T*^−1^ plot yields the activation energy 141 kJ mol^−1^, which is 65% larger than the value found in our previous work by using an advanced isoconversional method (Equations (16) and (17)). As established in the analysis of the simulated A3 and F2 data, more reasonable Arrhenius parameters are obtained while employing the ln*χ*(*T*)/*n* vs. *T*^−1^ plots. Indeed, the resulting *E* is 90 ± 3 kJ mol^−1^ and ln*A* is 20.9 ± 0.6. In agreement with the simulated results, the estimated *E* value is larger than the correct one by ~6%. In turn, the estimated ln*A* value is larger by ~12%.

## 7. Conclusions

Indubitably, the Ozawa–Avrami model can be applied to precisely determine the Avrami exponent as long as the process is established to obey the Avrami model. In the case of crosslinking polymerization, one cannot simply assume that the Avrami model applies. This is because the process can follow non-sigmoid (decelerating) reaction-order kinetics or more diverse autocatalytic kinetics. Only in the latter case the Avrami model may apply as a reasonable approximation. The most straightforward way to establish the Avrami model applicability is to perform at least one isothermal run and see whether its data can be fit by this model. An indirect way of testing this is to determine the value of conversion related to the rate (DSC) maximum, *α_p_*, under non-isothermal conditions. For the Avrami models, the respective value should be within the range 0.61–0.63 [55]. This criterion would help to differentiate the Avrami models from the second-order reaction model, for which *α_p_* is in the range 0.45–0.50. However, this criterion cannot distinguish the Avrami models from the first-order reaction model, for which *α_p_* is in the range 0.59–0.63. In addition, one should watch for a systematic dependence of the Avrami exponent on temperature as it can be a warning sign of the inapplicability of the Avrami model.

Concerning using the *χ*(*T*) function for determining the Arrhenius parameters, we have demonstrated that this function has a more complex temperature dependence than the regular rate constant, *k*(*T*). Yet, within certain limits, *χ*(*T*) can at best provide a mediocre approximation for *k*(*T*). However, if one is to use ln*χ*(*T*) in the Arrhenius plot, the values must be necessarily scaled by the values of the respective Avrami indexes (i.e., used as ln*χ*(*T*)/*n*). Without such a transformation, the errors in the activation energy can easily reach tens or even hundreds of percent. Additionally, not performing this transformation may produce breakpoints in the Arrhenius plots that confuse kinetic interpretations. Even if the transformation is performed, the Arrhenius plots of ln*χ*(*T*)/*n* still fail to produce the exact values of *E* and ln*A*.

Overall, the application of the Ozawa–Avrami model to non-isothermal polymerization is rather difficult to justify. It is limited to a single model, prone to computational artifacts, not very accurate, and not particularly easy to use. More importantly, there is a good number of time-proven kinetic techniques that are flexible, very accurate, and quite simple computationally [31]. Moreover, they have a solid track record of successful applications to crosslinking polymerization as well as many other processes.

## Figures and Tables

**Table 1:** The Ozawa–Avrami model parameters for the simulated A3 data.

T/K	n	ln [χ(T)/(K min^−1^)^n^]
394	3.00019	−1.24723
400	3.00009	0.21521
405	2.99992	1.4017
410	3.00002	2.56022
414	3.00005	3.46748

**Table 2:** The Ozawa–Avrami model parameters for the simulated F2 data.

T/K	n	ln [χ(T)/(K min^−1^)^n^]
380	0.95570	−1.69611
390	0.90559	−0.93423
400	0.82293	−0.2926
410	0.71248	0.21716
420	0.59298	0.60641
430	0.48454	0.90207

**Table 3:** The Ozawa–Avrami model parameters for the cyanate ester data.

T/K	n	ln [χ(T)/(K min^−1^)^n^]
553	3.1599	4.308
558	3.1596	4.8384
563	2.9663	4.9814
568	2.8487	5.3061
573	2.6340	5.4057

## Data Availability

Not applicable.

## References

[1] Schultz J. (2001). Polymer Crystallization.

[2] Mandelkern L. (2004). Crystallization of Polymers: Kinetics and Mechanisms.

[3] Djabourov M., Papon P. (1983). Influence of thermal treatments on the structure and stability of gelatin gels. Polymer.

[4] Huang X., Terech P., Raghavan S.R., Weiss R.G. (2005). Kinetics of 5α-Cholestan-3β-yl N-(2-Naphthyl)carbamate/n-Alkane Organogel Formation and Its Influence on the Fibrillar Networks. J. Am. Chem. Soc..

[5] Nasr P., Leung H., Auzanneau F.I., Rogers M.A. (2021). Supramolecular Fractal Growth of Self-Assembled Fibrillar Networks. Gels.

[6] Raposo M., Oliveira O.N. (2002). Adsorption of Poly(o-methoxyaniline) in Layer-by-Layer Films. Langmuir.

[7] Cestari A.R., Vieira E.F.S., Vieira G.S., Almeida L.E. (2006). The removal of anionic dyes from aqueous solutions in the presence of anionic surfactant using aminopropylsilica—A kinetic study. J. Hazard. Mater..

[8] Vargas A.M.M., Cazetta A.L., Kunita M.H., Silva T.L., Almeida V.C. (2011). Adsorption of methylene blue on activated carbon produced from flamboyant pods (Delonix regia): Study of adsorption isotherms and kinetic models. Chem. Eng. J..

[9] Serna-Guerrero R., Sayari A. (2010). Modeling adsorption of CO_2_ on amine-functionalized mesoporous silica. 2: Kinetics and breakthrough curves. Chem. Eng. J..

[10] Kole K., Das S., Samanta A., Jana S. (2020). Parametric Study and Detailed Kinetic Understanding of CO2 Adsorption over High-Surface-Area Flowery Silica Nanomaterials. Ind. Eng. Chem. Res..

[11] Zhang W.Z., Chen X.D., Luo W.-a., Yang J., Zhang M.Q., Zhu F.M. (2009). Study of Phase Separation of Poly(vinyl methyl ether) Aqueous Solutions with Rayleigh Scattering Technique. Macromolecules.

[12] Lo Nostro P., Giustini L., Fratini E., Ninham B.W., Ridi F., Baglioni P. (2008). Threading, Growth, and Aggregation of Pseudopolyrotaxanes. J. Phys. Chem. B.

[13] Fibich G. (2016). Bass-SIR model for diffusion of new products in social networks. Phys. Rev. E.

[14] Irzhak T.F., Mezhikovskii S.M., Irzhak V.I. (2008). The physical meaning of the Avrami equation in oligomer curing reactions. Polym. Sci. Ser. B.

[15] Pollard M., Kardos J.L. (1987). Analysis of epoxy resin curing kinetics using the Avrami theory of phase change. Polym. Eng. Sci..

[16] Lu M.G., Shim M.J., Kim S.W. (1998). The macrokinetic model of thermosetting polymers by phase-change theory. Mater. Chem. Phys..

[17] Lu M.G., Shim M.J., Kim S.W. (1998). Curing behavior of an unsaturated polyester system analyzed by Avrami equation. Thermochim. Acta.

[18] Kim S.-W., Lu M.-G., Shim M.-J. (1998). The Isothermal Cure Kinetic of Epoxy/Amine System Analyzed by Phase Change Theory. Polym. J..

[19] Lu M., Shim M., Kim S. (1999). Effect of filler on cure behavior of an epoxy system: Cure modeling. Polym. Eng. Sci..

[20] Ozawa T. (1971). Kinetics of non-isothermal crystallization. Polymer.

[21] Lu M.G., Shim M.J., Kim S.W. (1999). Dynamic DSC Characterization of Epoxy Resin by Means of the Avrami Equation. J. Therm. Anal. Calorim..

[22] Xin C., Yang X., Yu D. (2005). Non-isothermal Cure Kinetics of Polybenzoxazine/Carbon Fiber Composites by Phase Change Theory. Polym. Polym. Compos..

[23] Hong Zhang X., Qin Min Y., Zhao H., Mei Wan H., Rong Qi G. (2006). Novel nitrogen-containing epoxy resin. II. Cure kinetics by differential scanning calorimetry. J. Appl. Polym. Sci..

[24] Janeczek H., Siwy M., Schab-Balcerzak E. (2009). Polymers based on N,N-diglycidylaniline. I. Investigations of the curing kinetics by dynamic differential scanning calorimetry measurements. J. Appl. Polym. Sci..

[25] Liu Q.Y., Chen J.B., Liu S.M., Zhao J.Q. (2012). Dynamic cure kinetics of epoxy resins using an amine-containing borate as a latent hardener. Polym. Int..

[26] Wang W., Di N.Y., Cao W.R., Liu X.D., Yao J.M. (2015). Cure kinetics of epoxy resin using 1,2,4,5-benzenetetracarboxylic acid/2-ethyl-4-methylimidazole salt as a latent hardener. Mater. Res. Innov..

[27] Cao H., Liu B., Ye Y., Liu Y., Li P. (2019). Reconstruction of the Microstructure of Cyanate Ester Resin by Using Prepared Cyanate Ester Resin Nanoparticles and Analysis of the Curing Kinetics Using the Avrami Equation of Phase Change. Appl. Sci..

[28] Prime R.B., Turi E.A. (1997). Thermosets. Thermal Characterization of Polymeric Materials.

[29] Yousefi A., Lafleur P.G., Gauvin R. (1997). Kinetic studies of thermoset cure reactions: A review. Polym. Compos..

[30] Vyazovkin S., Sbirrazzuoli N. (1999). Kinetic methods to study isothermal and nonisothermal epoxy-anhydride cure. Macromol. Chem. Phys..

[31] Vyazovkin S., Burnham A.K., Criado J.M., Perez-Maqueda L.A., Popescu C., Sbirrazzuoli N. (2011). ICTAC Kinetics Committee recommendations for performing kinetic computations on thermal analysis data. Thermochim. Acta.

[32] De Keer L., Kilic K.I., Van Steenberge P.H.M., Daelemans L., Kodura D., Frisch H., De Clerck K., Reyniers M.-F., Barner-Kowollik C., Dauskardt R.H. (2021). Computational prediction of the molecular configuration of three-dimensional network polymers. Nat. Mater..

[33] Borchardt H.J., Daniels F. (1957). The Application of Differential Thermal Analysis to the Study of Reaction Kinetics1. J. Am. Chem. Soc..

[34] Šesták J. (2019). Ignoring heat inertia impairs accuracy of determination of activation energy in thermal analysis. Int. J. Chem. Kinet..

[35] Vyazovkin S. (2020). How much is the accuracy of activation energy affected by ignoring thermal inertia?. Int. J. Chem. Kinet..

[36] Vyazovkin S. (2020). Activation Energies and Temperature Dependencies of the Rates of Crystallization and Melting of Polymers. Polymers.

[37] Bruijn T.J.W.D., Jong W.A.d., Berg P.J. (1981). Kinetic parameters in Avrami-Erofeev type reactions from isothermal and non-isothermal experiments. Thermochim. Acta.

[38] Yinnon H., Uhlmann D.R. (1983). Applications of thermoanalytical techniques to the study of crystallization kinetics in glass-forming liquids, part I: Theory. J. Non-Cryst. Solids.

[39] Fatemi N., Whitehead R., Price D., Dollimore D. (1986). Some comments on the use of Avrami-Erofeev expressions and solid state decomposition rate constants. Thermochim. Acta.

[40] Khanna Y.P., Taylor T.J. (1988). Comments and recommendations on the use of the Avrami equation for physico-chemical kinetics. Polym. Eng. Sci..

[41] Brown M.E., Galwey A.K. (1989). Arrhenius parameters for solid-state reactions from isothermal rate-time curves. Anal. Chem..

[42] Jackson K.A. (2004). Kinetic Processes. Crystal Growth, Diffusion, and Phase Transitions in Materials.

[43] Vyazovkin S. (2018). Nonisothermal crystallization of polymers: Getting more out of kinetic analysis of differential scanning calorimetry data. Polym. Cryst..

[44] Haudin J.M., Billon N. (1992). Solidification of semi-crystalline polymers during melt processing. Progr. Colloid Polym. Sci..

[45] Hieber C.A. (1995). Correlations for the quiescent crystallization kinetics of isotactic polypropylene and poly(ethylene terephthalate). Polymer.

[46] Sajkiewicz P., Carpaneto L., Wasiak A. (2001). Application of the Ozawa model to non-isothermal crystallization of poly(ethylene terephthalate). Polymer.

[47] Zhang Z., Xiao C., Dong Z. (2007). Comparison of the Ozawa and modified Avrami models of polymer crystallization under nonisothermal conditions using a computer simulation method. Thermochim. Acta.

[48] Vyazovkin S. (2015). Isoconversional Kinetics of Thermally Stimulated Processes.

[49] Senum G.I., Yang R.T. (1977). Rational approximations of integral of Arrhenius function. J. Therm. Anal..

[50] Flynn J.H. (1997). The ‘Temperature Integral’—Its use and abuse. Thermochim. Acta.

[51] Doyle C.D. (1961). Kinetic analysis of thermogravimetric data. J. Appl. Polym. Sci..

[52] Vyazovkin S. (2001). Modification of the integral isoconversional method to account for variation in the activation energy. J. Comput. Chem..

[53] Vyazovkin S. (2021). Determining Preexponential Factor in Model-Free Kinetic Methods: How and Why?. Molecules.

[54] Galukhin A., Nosov R., Taimova G., Islamov D., Vyazovkin S. (2021). Synthesis and Polymerization Kinetics of Novel Dicyanate Ester Based on Dimer of 4-tert-butylphenol. Macromol. Chem. Phys..

[55] Gao X., Chen D., Dollimore D. (1993). The correlation between the value of α at the maximum reaction rate and the reaction mechanisms: A theoretical study. Thermochim. Acta.

